# Spin-related excited-state phenomena in photochemistry

**DOI:** 10.1093/nsr/nwae244

**Published:** 2024-07-18

**Authors:** Chuang Zhang, Chen Ye, Jiannian Yao, Li-Zhu Wu

**Affiliations:** Key Laboratory of Photochemistry, Beijing National Laboratory for Molecular Sciences, Institute of Chemistry, Chinese Academy of Sciences, Beijing 100190, China; Key Laboratory of Photochemical Conversion and Optoelectronic Materials, Technical Institute of Physics and Chemistry, Chinese Academy of Sciences, Beijing 100190, China; Key Laboratory of Photochemistry, Beijing National Laboratory for Molecular Sciences, Institute of Chemistry, Chinese Academy of Sciences, Beijing 100190, China; Key Laboratory of Photochemical Conversion and Optoelectronic Materials, Technical Institute of Physics and Chemistry, Chinese Academy of Sciences, Beijing 100190, China

**Keywords:** electron spin, excited state, magnetic field, luminescence, photocatalysis

## Abstract

The spin of electrons plays a vital role in chemical reactions and processes, and the excited state generated by the absorption of photons shows abundant spin-related phenomena. However, the importance of electron spin in photochemistry studies has been rarely mentioned or summarized. In this review, we briefly introduce the concept of spin photochemistry based on the spin multiplicity of the excited state, which leads to the observation of various spin-related photophysical properties and photochemical reactivities. Then, we focus on the recent advances in terms of light-induced magnetic properties, excited-state magneto-optical effects and spin-dependent photochemical reactions. The review aims to provide a comprehensive overview to utilize the spin multiplicity of the excited state in manipulating the above photophysical and photochemical processes. Finally, we discuss the existing challenges in the emerging field of spin photochemistry and future opportunities such as smart magnetic materials, optical information technology and spin-enhanced photocatalysis.

## Teaser

This review provides an overview on the concept of spin photochemistry, and summarizes some recent advances on spin-related excited state phenomena in terms of magnetism, luminescence and reactivity.

## INTRODUCTION

Electron spin is an intrinsic quantum property of angular momentum and plays an essential role in chemical sciences. The electron configuration of any material obeys the Pauli exclusion principle, i.e. an orbital can hold a maximum of two electrons—one is spin-up and the other is spin-down. The outer-shell electrons not only determine the chemical properties of materials, but are also related to their magnetic properties according to the contribution from net electron spins. This is because the magnetic moment of electron spin is significantly larger than that of nuclear spin or orbital angular momentum. Spin is thus correlated with numerous chemical processes through electron transfer/rearrangement during reactions. Transition metals are extensively used for their magnetic properties and spin-related phenomena, due to the existence of unpaired electrons in their outermost d orbitals. Radicals are another important species for investigating spin-related properties, as their unpaired electrons bring chemical reactivities and make them reaction intermediates in many chemical processes. One of the most mentioned examples is magnetoreception, or the so-called biocompass [1], in which the biochemical reactions involving radicals are influenced by Earth's magnetic field. One hypothesis describes that a pair of radicals is generated by the excitation of light-sensitive molecules and the reaction pathways of such short-lived radical pairs depend on the interaction of Earth's field with these transient spin species. Another example is the photosynthetic reaction center [2] in which radical–ion pairs are formed by harvesting the photon energy and are essential for the subsequent biochemical reactions. It implies that the absorption of photons and subsequently the electronic transitions would change both the electron configuration and the spin configuration of materials, leading to the observation of spin-related phenomena and properties in excited-state chemistry [3,4].

**Figure 1.**
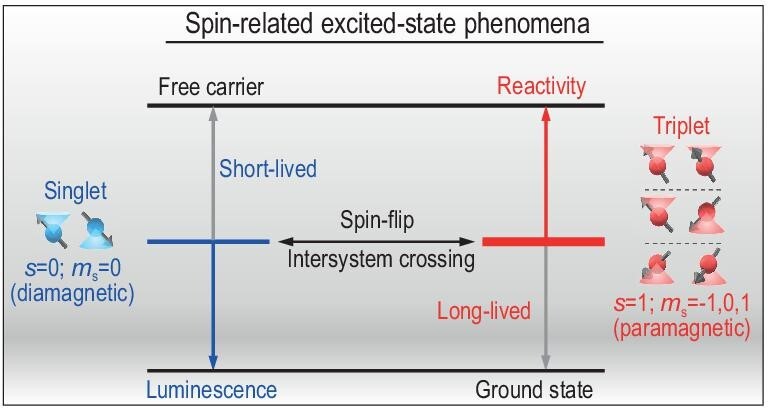
Schematic illustration of spin-related phenomena based on the spin-flip conversion between singlet and triplet, which contribute differently to the excited-state properties (magnetism, luminescence, reactivity, etc.).

The excited state empowers studies of photochemistry in a variety of applications, including energy harvesting, organic optoelectronics, photocatalysis, bioimaging and so on. The spin multiplicity of electrons is naturally involved in the excited state because the total spin angular momentum (*s*) may be altered when one of the electrons is excited to the upper level, i.e. both singlet (*s* = 0; *m*_s_ = 0) and triplet (*s* = 1; *m*_s_ = −1,0,1) can be obtained for the two-particle system [5], as shown in Fig. 1. It should be mentioned that they are not representatives with exact proportions; in fact, the singlet/triplet shows a ratio of 1/3 under electron injection, while only the singlet is optically accessible in most cases. Although the spin is usually conserved during optical transitions, singlet may convert into triplet excited states through spin-flipped intersystem crossing. As there is a net spin for triplet, they are considered as transient paramagnetic species that give rise to magnetic property of excited-state materials. More interestingly, singlet and triplet may show very different photophysical and photochemical properties due to the spin-selective relaxation to the ground state that is usually spin-singlet. For instance, singlet is relatively short-lived and more emissive (fluorescence) than triplet in organic chromophores, unless the spin-forbidden transition of a long-lived triplet excited state becomes semi-allowed (phosphorescence) due to spin–orbit interaction. The interplay between spin sublevels of the excited state would lead to the observation of magnetic-field effects on luminescence [6], which are considered to be excited-state magneto-optical effects. In addition, short-lived singlet and long-lived triplet may show quite different reactivities, e.g. triplet is sensitive to oxygen, which is a ground-state triplet molecule. The photogeneration of reactive oxygen species [7], widely used in photodynamic therapy, is dependent on the triplet formation of a photosensitizer, and is also proven to be sensitive to external magnetic fields [8]. It is a remarkable fact that the spin interaction shows profound influences on some of the photochemical processes, although its strength is several orders of magnitude smaller than the thermal energy at room temperature. This means that the conservation of angular momentum is not in conflict with the conservation of energy; both of these conservation laws should be taken into account in understanding excited-state properties. Therefore, it requires further attention on the photochemistry phenomena involving spin sublevels of the excited state and it is necessary to explore their potentials in both overcoming the limitations in conventional applications (e.g. solar energy conversion) [9] and discovering unexpected advantages in emerging techniques (e.g. information technology) [10].

**Figure 2.**
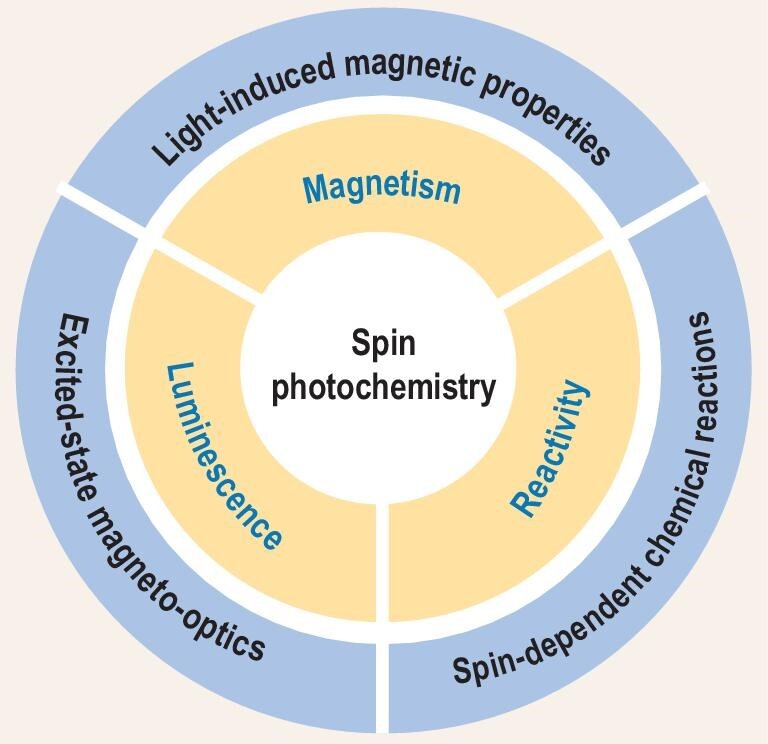
Schematics for the research topics of spin photochemistry included in this review.

In this article, our goal is to present and discuss the concept of spin photochemistry as an important branch of the fascinating field of spin chemistry, in which the spin-related physical and chemical properties originate from the spin multiplicity of the photogenerated excited state. The history of spin photochemistry is first introduced to show both the experimental and the theoretical fundamentals for the spin manipulation of the excited state, especially by means of applying an external magnetic field, and consequently the influence on its magnetic, optical and chemical properties. The spins of excited states are related to magnetism (‘spin’) and their spin-selective transitions are related to luminescence (‘photo-’) and reactivity (‘chemistry’). Therefore, the recent advances in spin photochemistry, including (i) light-induced magnetic properties, (ii) excited-state magneto-optical effects and (iii) spin-dependent photochemical reactions, are presented, as illustrated in Fig. 2. Specifically, photogenerated spin species bring the non-permanent paramagnetic centers; magneto-optical effects are observed on the luminescence from spin-dependent transitions; photoreactions involving radical intermediates show the spin-selective chemical reactivity of the excited state. Finally, our personal insights and the future opportunities are given, as the research on spin photochemistry is still in its infancy, with an emphasis on the potential applications of smart materials for robotics and optical spin control for information science and for chemical/biochemical synthesis. We hope that this review may provide a useful reference to both experimentalists and theorists in photochemistry, as well as to the community of chemists and physicists who are working on the areas of organic spintronics, optoelectronics, radicals, luminescent materials and so on.

## HISTORY OF SPIN PHOTOCHEMISTRY STUDIES

In fact, spin photochemistry—referring to photogenerated spin species, magnetic-field effects and other spin-related phenomena in photochemistry studies—is not an entirely new concept. In the early 1980s, Turro and Kraeutler presented a description of using the theory of intersystem crossing in radical pairs to interpret magnetic-field and magnetic-isotope effects on organic photochemical reactions [11]. It was found that the paramagnetic species could be photogenerated and detected by using magnetic resonance techniques. Therefore, the external magnets or the nuclear magnets could allow the collapse of the spin conservation rule for chemical reactions, which is utilized to control the efficiency and selectivity of cage reactions of radical pairs. Even before that, Merrifield summarized the observation of magnetic-field effects on the delayed fluorescence in molecular crystals from the fusion of triplet excitons [12], which could be attributed to the influence of the external field on the spin wave functions of triplets together with the existence of spin selection rules for their interactions. The theory behind these phenomena indicates that the rate of spin-selective processes involving triplet exciton interactions is magnetic-field-sensitive, and it consequently influences the delayed fluorescence upon the triplet-to-singlet conversion. Thermodynamically, it could be assumed that the rate of chemical processes should be changed by a magnetic field if the initial–final states are either diamagnetic–paramagnetic or paramagnetic–diamagnetic. However, the magnetic contribution to the Gibbs free enthalpy of the reaction is estimated to be ∼0.05 J mol^−1^ in a field of 1 T, which corresponds to a change in the equilibrium constant by a factor of ∼10^−5^ at room temperature. In most cases, the magnetic energies are negligible compared with the thermal energies in chemical reactions. Therefore, the significant magnetic-field effects on the rates and/or efficiencies of chemical processes must involve quantum mechanical phenomena (spins) that are beyond the scope of classical thermodynamics. The underlying mechanism of spin-1/2 (radical) pairs is discovered after the understanding of chemically induced nuclear and electronic spin-polarization phenomena in reactions. Radical pairs in singlet electronic states are more likely to form combination products than the pairs in triplets, while the eigenfunctions of spin Hamiltonian are usually not the pure spin states and undergo coherent evolution influenced by an external field. It subsequently allows the experimental observation of magnetokinetics, i.e. magnetic-field effects on the kinetics of chemical reactions, which was comprehensively reviewed by Steiner and Ulrich [13].

The concept of spin photochemistry is a combination of spin physics and the chemical reactivity of excited states, so both chemical and physical phenomena should be involved and are closely related from the theoretical point of view. Wigner introduced the conservation of spin angular momentum in chemical reactions [14]. A chemical process is defined as ‘spin-allowed’ if the spin angular momentum space spanned by the reactants overlaps with that spanned by the product; otherwise, it should be ‘spin-forbidden’. A simple example is to produce close-shell diamagnetic species through the reactions of the intermediate of radical pairs, which has four spin states including a singlet and three triplet states. Wigner's rule states that, in principle, only the singlet of radical pairs can convert to the products and give a maximum of 25% yield. For example, it is consistent with the maximum internal quantum efficiency (25%) of fluorescent (singlet emission) organic light-emitting diodes (OLEDs), which originates from the radiative recombination of spin-singlet electron–hole pairs. It also implies that the photochemical reaction can be controlled by spin-state manipulation according to its spin dependence, because the transition between spin states can be decelerated or accelerated by a static and/or oscillating external magnetic field [15].

In the early 2000s, the blooming of two emerging fields, organic electronics and spin-based electronics (spintronics), gave rise to a new cross-field of organic spintronics [16]. It not only defines spin-based technology using organic semiconductors, but also describes studies on fundamental microscopic spin-dependent processes in molecular materials [17]. The original idea was to improve the efficiency of OLEDs by injecting spin-polarized electrons/holes to form a large portion of singlets. In addition to that, the phenomenon of spin photochemistry was ‘rediscovered’ in organic-based devices, i.e. the intrinsic resistance in some organic semiconductors significantly varies in a small magnetic field. It was called ‘organic magnetoresistance’, in which the dissociation and charge reaction in excited states provides a convenient pathway to tune the magnetoresistance in organic semiconductors [18]. Importantly, the injection of spin-polarized current into organic semiconductors was demonstrated by observing the giant magnetoresistance of an organic-based spin valve [19] as an important milestone in the development of organic spintronics. The performance of such spin-optoelectronic devices can thus be controlled by manipulating the spin states within the organic active layer in devices. For instance, the electroluminescence is sensitive to the spin polarization of the injected carriers in spin-OLEDs [20], while the photovoltaic response is modified under the application of a small field in a spin-photovoltaic device [21]. These advances in organic spintronics in past decades have been reviewed elsewhere and are not our emphasis here. It should be noted that these spin manipulation mechanisms [22] revealed in spin physics studies would also benefit the development of spin photochemistry.

A new era for spin-related studies on excited states has arrived in the past few years, as the topic has attracted much attention from the interdisciplinary research community. Some unanswered questions have been raised and inspire further theoretical and experimental investigations [23,24], especially for in-depth understanding in terms of spin dynamics on photochemical processes, such as intersystem crossing and charge-transfer reactions. Furthermore, spin dynamics is essential for quantum mechanical control over reactions involving transient radical species, which is completely different from the conventional reaction control by thermal energy. It is not only important for the development of fundamental theories in photochemistry, but also useful for designing chemical compounds towards light-responsive magnetic materials and multifunctional opto-spintronics devices [25], as well as for overcoming the efficiency limitations in solar energy conversion and storage. Some of the recent breakthroughs related to spin-related excited-state phenomena in photochemistry are summarized below.

## LIGHT-INDUCED MAGNETIC PROPERTIES

Upon the absorption of photons by the optically active materials, the electronic transition would lead to the decoupling of paired electrons and may consequently change the total spins in the system. Electron-spin resonance (ESR) spectroscopy is sensitive for the detection of paramagnetic species from unpaired electrons and is used to investigate the nonzero spins of electronic states in both organic and inorganic materials. For the generation of electron–hole pairs by light absorption, ESR spectroscopy is modified to involve the optical excitation of the sample during the measurements, namely the light-induced ESR technique, to study the properties of paramagnetic species from the charge-transfer process that subsequently occurs after light absorption. Light-induced ESR has been widely used as a powerful tool in the areas of photovoltaics and photocatalysis [26]. Notably, the singlet excitons directly created by light absorption are invisible due to their spin zero nature; however, the electron/holes generated by charge separation at the donor/acceptor interface as well as the triplet excitons may show up in light-induced ESR spectroscopy.

These light-induced paramagnetic species may also change the magnetic properties of optically active materials, and the change in magnetic susceptibility upon light excitation is usually called photomagnetism [27]. This effect can be either transient, which is associated with the magnetic properties of short-lived excited states, or semi-permanent, which is based on photochemical reactions such as photogenerated radicals. Besides, coordination compounds are particularly interesting for phase-transition photomagnetic materials, in which the selection of magnetic metal ions and organic ligands provides a way of controlling their electronic states and spin–spin interactions by light irradiation [28–30]. The exploration of spin-nonzero excited states and light-induced changes in magnetic susceptibility offer a promising approach for the design of intelligent materials that can respond to both magnetic fields and light exposure [31].

### Light-induced ESR

**Figure 3.**
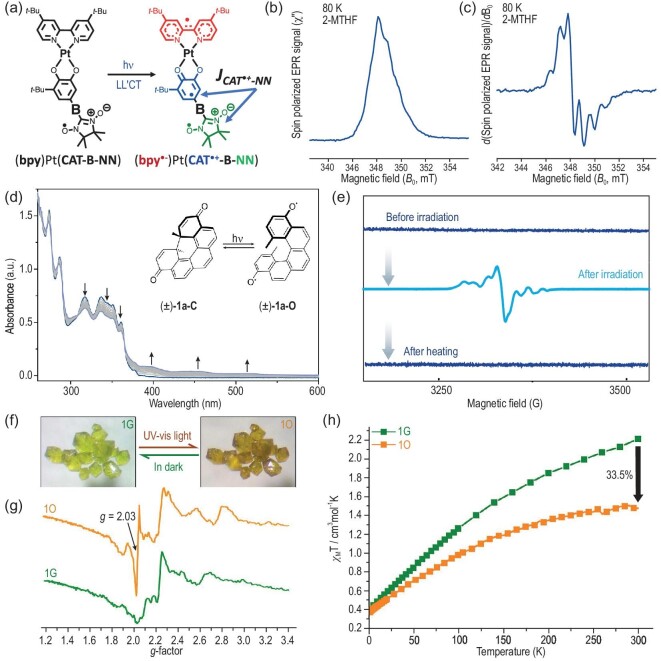
(a) Chemical structure of the radical-appended Pt complex in which the ligand-to-ligand charge transfer (LL′CT) under photoexcitation yields a three-spin excited state with bridge(B)-modulated magnetic exchange. (b) Light-induced spin-polarized ESR spectrum and (c) its first derivative measured at 4 μs after the laser excitation at 532 nm from the radical-appended Pt complex. Reproduced from Ref. [33] with permission. (d) UV–vis absorption spectra of compound **1a** recorded every 60 s during UV light irradiation at 77 K. (e) ESR spectra taken from **1a** before and after irradiation and thermal back reaction. Reproduced from Ref. [41] with permission. (f) Photochromism of an Eu-Fe complex (marked as 1G and 1O) in its bulk crystals at room temperature. (g) ESR spectra of Eu–Fe complex at room temperature. (h) Magnetic susceptibility versus temperature for Eu–Fe complex at *H* = 5000 Oe. Reproduced from Ref. [43] with permission.

In the simplest scenario for light-induced ESR measurements, all electrons are paired in a diamagnetic ground state. The emergence of ESR signals upon optical excitation is then attributed solely to the photogeneration of unpaired spins. However, the situation becomes complicated and more interesting when the ground state is not spin-neutral. Recently, transient ESR spectroscopy has been adopted by Kirk *et al.* to probe photo-induced electron-spin polarization in the Pt(II) complex incorporating nitroxide radicals [32], as shown in Fig. 3a. They achieved spin polarization in electronic ground states using optical excitation in these radical-appended complexes. Furthermore, they introduced two radicals covalently attached to the complex, which are exchange-coupled spin pairs [33]. A light-induced spin-polarized time-resolved EPR spectrum (Fig. 3b) and its first derivative (Fig. 3c) were collected 4 μs after the laser pulse and revealed transient spin polarization upon optical excitation of the biradical complex with exchange-coupled radicals. The fast polarization and initialization of two exchange-coupled spins were realized after single-photon excitation. This holds the potential for fast optical readout of spin states using emissive donor–acceptor chromophores. Another example by Gorgon *et al.* is the covalent bonding of radicals to the anthracene molecule to generate a high-spin state suitable for optical readout [34]. Optical excitation in the radical–anthracene structures facilitates the obtainment of spin-polarized states by adopting the doublet energy level of radicals for light absorption and emission. The energy resonance between doublet and triplet levels in the excited-state manifold with small energy offsets provides a pathway for the photogeneration of high-spin configurations on their hybridized excited states. ESR measurements not only enable the quantification and temporal characterization of photogenerated spins, but also offer an ideal platform to manipulate these spin states combined with optical write-in and/or readout methods.

Chirality-induced spin selectivity (CISS) refers to the phenomenon in which spin-polarized electron transport through chiral molecules is dependent on the specific chirality of the molecule [35]. Although CISS has been regarded as a promising method for spin polarization, it should be noted that chirality itself does not introduce paramagnetic species in the compounds and thus cannot be detected by using ESR. Very recently, the role of CISS has been investigated in the spin dynamics of donor-chiral bridge-acceptor molecules using time-resolved ESR spectroscopy under laser pulses by Eckvahl *et al*. [36]. By analysing the lineshape of the ESR signal, they concluded that the photo-induced electron transfer from donor to acceptor through the chiral bridge undergoes CISS. The donor–acceptor radical pairs are photogenerated and spin-correlated, and CISS mixes their triplet character into the initial singlet after photoexcitation. In addition to the spin-1/2 photocarriers, triplets with spin = 1 are another kind of excited-state species that can be detected by using ESR. Light-induced and angle-resolved ESR techniques were employed in tetracene crystals to investigate the spin properties of photogenerated triplets through either the intersystem crossing from singlets or the dissociation of entangled triplets during singlet fission [37]. Combining time-resolved optical and spin resonance methods is useful to probe the dynamics of spin diffusion and transfer in a variety of materials. Co-crystals composed of donor and acceptor molecules are suitable for controlling the photophysical and magnetic characteristics of triplet excitons. Time-resolved pulse-ESR spectroscopy was utilized to investigate the spin properties of excited states in co-crystals, in which spin-polarized triplet excitons are formed following the selective photoexcitation of charge-transfer optical transitions and consequently the sub-nanosecond intersystem crossing on the charge-transfer state [38]. In these single crystals, the EPR line widths and resonant field positions exhibit high sensitivity to the crystal orientation relative to the applied magnetic field. Furthermore, the light-induced triplet–triplet electron resonance spectroscopy developed by Bertran *et al.* can measure the dipolar interaction between two photogenerated triplet states at the nanometer scale [39]. This technique is useful for determining the distance and angular distributions between two triplet moieties that lack stable paramagnetic centers and is particularly suitable for some biological photochemical systems such as light-harvesting proteins.

One additional case for light-induced ESR is the photochemical reactions that produce free radicals or modify pre-existing radicals upon exposure to light. It also leads to a subsequent change in ESR signals from light-induced radicals in chemical compounds. The use of light to release radicals is considered a rational synthesis strategy of light-controlled radical polymerizations, including intramolecular photochemical processes and photoredox processes [40]. Controlling the electronic spin state of chemical compounds through an external stimulus is of particular interest. For instance, the spin states of photochromic molecule **1a** in open (**1a-O**) and close (**1a-C**) forms (Fig. 3d) can be photochemically switched [41]. As shown in Fig. 3e, the open-form **1a-O** is diamagnetic and ESR-silent at cryogenic temperature, while the close-form **1a-C** is obtained after light irradiation and becomes paramagnetic (EPR-active). A thermal back reaction could occur at room temperature and convert it immediately into the pure ESR-silent form **1a-C**. Light-induced radicals also offer possibilities for changing the magnetic susceptibility of optically active materials.

### Light-induced change in magnetic susceptibility

Magnetism is a collective phenomenon at the macroscopic scale involving the cooperation of many microscopic magnetic moments in chemical compounds. Magnetic bistability, in which the magnetic susceptibility varies upon the external stimuli, is of great interest in data storage, magnetic sensing, spintronics and so on. For instance, a nickel complex serves as a molecular spin switch that is magnetically bistable at room temperature [42]. Irradiation using 500 nm of light induces an electronic rearrangement from a diamagnetic to a paramagnetic state, and the process is reversible on irradiation with 435 nm of light. The photomagnetism of nickel complex is very robust even after several thousand cycles in a dilute solution under air. Cai *et al.* report the photochromism and photomagnetism of 3d–4f hexacyanoferrates at room temperature [43]. In the bulk crystal of an Eu–Fe complex (Fig. 3f), the light irradiation triggers a green(G)-to-orange(O) color change due to the photo-induced electron transfer from Eu crown to Fe counterpart, as revealed by the changes in the ESR signals (Fig. 3g). The excited-state process yields long-lived charge-separated species in air and weakens the magnetic susceptibility significantly in the solid state, as shown in Fig. 3h. The polycyanometallate compound is both photochromic and photomagnetic, implying opportunities in manipulating the spin transition of metal ions to achieve switchable multifunctions under light irradiation in organic–inorganic hybrid materials [44]. The photomagnetism and photo-induced spin transition take advantage of high spatial and temporal resolutions in photoirradiation used in switching the high- and low-spin states.

The magnetic interactions, and consequently the magnetic order, are essential for ferromagnetic materials. The sign and strength of antiferromagnetic/ferromagnetic interactions are determined by the geometry of chemical bonds, and thus the *in situ* fine-tuning of magnetic interactions is usually difficult. Náfrádi *et al.* demonstrated optically switched magnetism in an emerging hybrid semiconductor, namely the photovoltaic perovskite CH_3_NH_3_(Mn:Pb)I_3_ [45]. This Mn-substituted perovskite shows a combination of high-efficiency generation of photocarriers and ferromagnetism at cryogenic temperature, and optical control of the magnetism is achieved by controlling the competition of magnetic interactions between itinerant and localized electrons under visible-light illumination. The Mn^2+^–Mn^2+^ magnetic coupling and its effect on optical properties were further investigated in the CsMnCl_3_ perovskite by Zhu *et al*. [46]. Photomagnetism was observed in the pristine CsMnCl_3_ but not in its hydrate due to the lack of a Mn^2+^–Mn^2+^ pair, and the magnetic exchange coupling of the Mn^2+^–Mn^2+^ pair shortens the decay lifetime of the photoluminescence associated with Mn^2+^ centers. The interaction between spin and photon was also found in organic chiral materials without metal centers [47]. The reversible spin-optical interface leads to a series of excited-state magneto-optical effects, especially in luminescent materials.

## EXCITED-STATE MAGNETO-OPTICAL EFFECTS

Magnetic-optical effects are conventionally referred to as the Faraday effect or the magneto-optical Kerr effect, in which the polarization of light transmission rotates in a medium that is magnetized by an external field. In comparison, the spin-related properties of excited states may expand the definition of magnetic-optical effects in optically active materials. Optical transitions obey the spin selection rule across the electronic energy levels and enable conversion between the spin of electrons and the polarization of light in the absorption or emission of photons. For instance, circularly polarized light absorption/emission has been observed in direct-gap III–V semiconductors [48], monolayer transition metal dichalcogenides [49], phosphorescent metal complexes [50] and organic–inorganic hybrid materials [51]. Circularly polarized emission can be achieved by either Zeeman spin splitting under a high magnetic field at a low temperature or optical/electronic spin injection at room temperature. The competition between radiative decay and spin relaxation rates determines the degree of circular polarization in the light emission. Field-induced circular polarization requires thermal equilibrium on spin sublevels that undergo energy splitting in which a fast spin relaxation is preferred to reach the equilibrium state. In contrast, spin-injected circularly polarized emission is from a non-equilibrium state of spin polarization and a faster radiative decay from the recombination of spin-polarized carriers is crucial.

Overall, spin manipulation of the excited state allows the observation of magneto-optical effects on the polarization in absorption/emission rather than light transmission. More interestingly, the intensity of photoluminescence can also be dependent on the magnetic field—so-called magneto-photoluminescence, which is predominately related to excited states in photochemical processes. The underlying mechanism is that the bright and dark states usually have different spin configurations and the spin conversion between them is influenced by applying an external field. Similar effects, such as magneto-photoconductance and magneto-electroluminescence, have also been observed in organic semiconductors and these magnetic-field effects on opto-electronic properties have been reviewed previously [52–55]. The excited state is considered as a spin pair in which the configuration can be altered by an external magnetic field through the hyperfine interaction, the spin–orbit interaction and/or the spin precession with different g-factors. Here, we focus on magneto-photoluminescence in luminescent radicals involving doublet ground states and magneto-optical effects via triplet–triplet pairs (high-spin excited states), which offer opportunities for the design and fabrication of state-of-the-art magneto-optoelectronic devices.

### Magneto-photoluminescence in luminescent radicals

Spin manipulation of excited states is essentially important for OLEDs in display and lighting technologies. Most luminescent molecules are closed-shell and the ground state is spin-0 singlet. The spin selection rule allows only optical transitions between the singlet ground state and the singlet excited state, while the triplet excited state is optically forbidden (dark) and obtained usually through intersystem crossing. In the luminescent radicals that belong to a family of open-shell molecules, the outermost molecular orbital is occupied by one unpaired electron and the spin configuration of the ground state is doublet. Similarly, the first excited state has one unpaired electron on a singly occupied molecular orbital and its spin quantum number remains at 1/2. Since both the ground and first excited states of luminescent radicals are doublet, the doublet–doublet optical transition, termed as doublet emission, is spin-allowed [56]. The doublet character of luminescent radicals can be used in harvesting the dark triplet excited state through energy transfer. Li *et al.* studied the energy transfer channels between singlet, triplet and doublet excitons, and explored the singlet–doublet and triplet–doublet energy transfer mechanisms using organic radicals [57]. Magneto-optical effects indicate that dark triplet states are removed in the OLEDs incorporated with fluorescent radical emitters through the rapid triplet–doublet energy transfer, showing the role of unpaired electron-spin properties in radical-based light-emitting materials and devices.

**Figure 4.**
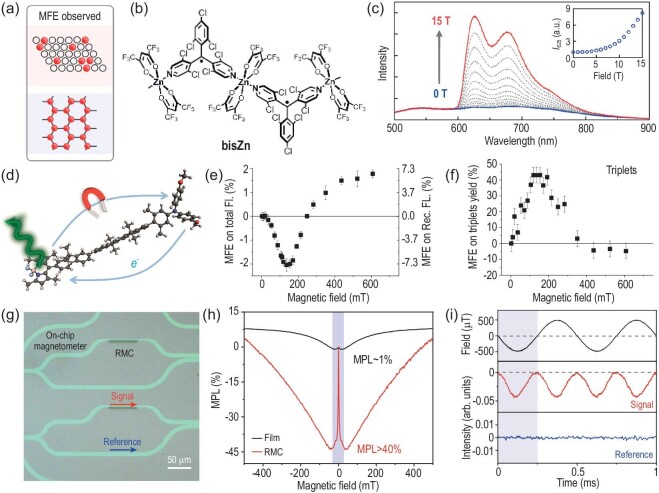
(a) Schematic illustration of the magnetic-field effect (MFE) observed in radical doped crystals and radical-based coordination polymers. (b) Molecular structure of the radical-based coordination polymer, **bisZn**. (c) Emission spectra of **bisZn** measured at 4.2 K under a magnetic field of ≤15 T. Inset indicates the field dependence of the emission intensities recorded at 625 nm. Reproduced from Ref. [59] with permission. (d) Schematic illustration of the magneto-optical effects observed in rigid donor–bridge–acceptor molecules. (e) MFE on total fluorescence and recombination fluorescence as a function of field strength of ≤600 mT. The fraction of recombination fluorescence in total fluorescence is 0.27. (f) MFE on the production of triplets as a function of field strength of ≤600 mT. Reproduced from Ref. [64] with permission. (g) Optical microscopy image of the on-chip magnetometer based on rubrene microcrystals. (h) Magneto-photoluminescence (MPL) curves measured from rubrene film and microcrystal (RMC) up to a field strength of 500 mT. (i) Response of rubrene microcrystal magnetometer in signal and reference under an applied alternative field. Reproduced from Ref. [72] with permission.

Spin-allowed doublet–doublet transition not only exhibits unique luminescence properties, but is also sensitive to external stimuli and environments including magnetic fields. Kimura *et al.* investigated the monomer and excimer emission properties of a stable radical (3,5-dichloro-4-pyridyl)bis(2,4,6-trichlorophenyl)methyl (PyBTM) by doping it into the molecular crystal of a H-PyBTM host and found that the intensity ratio of these two emission bands at cryogenic temperature was modulated drastically by applying a magnetic field of ≤18 T [58]. The excited state of the radical monomer is doublet and it can be coupled with a neighboring radical monomer in the ground state to produce an excimer of a radical pair that is either singlet or triplet. Due to the different spin characteristics of excited states for a monomer and an excimer, the applied magnetic field influences the spin conversion of excited states and increases the monomer emission but decreases the excimer emission. Interestingly, magneto-photoluminescence is observed only in moderately (4%–10%) doped samples but not in lightly (0.1%–1%) doped crystals. It implies that the aggregated radicals (Fig. 4a) and their spin sublevel populations are critical for the observation of large magneto-optical effects. Kimura *et al.* further developed radical-based coordination polymers, such as 1D **bisZn** (Fig. 4b) and 2D **trisZn**, and they investigated the photoluminescence from these radical-based coordination polymers under an external field [59]. As shown in Fig. 4c, the photoluminescence of **bisZn** is negligible emission at zero field and is significantly enhanced by an applied magnetic field of ≤15 T at cryogenic temperature. Note that the magneto-optical effect is absent in the condensed radical molecules, which indicates that the exchange interaction between radicals is weakened through the spatial separation of radicals in radical-based coordination polymers. It thus allows magnetic fields to modulate the populations on spin sublevels of radicals in their spin-nonzero ground states.

Open-shell singlet diradicals are important building blocks for functional molecular materials; Abdurahman *et al.* designed a luminescent diradical and found that its singlet ground state could be thermally excited to triplet due to the small singlet–triplet energy gap [60]. In addition, they observed large magneto-photoluminescence of 210% under an applied field of 7 T at cryogenic temperature. The interplay between the singly occupied molecular orbital and the lowest unoccupied molecular orbital enables spin manipulation on ground states and consequently the magnetic-field effects on doublet optical transitions. Matsuoka *et al.* investigated the photophysical and magneto-optical properties of a luminescent molecule containing two spatially proximate radicals [61]. The diradical molecule shows an obvious change in its emission spectra under a magnetic field of ≤14.5 T at cryogenic temperature, while the magneto-photoluminescence is not observed in the corresponding monoradical counterpart. These results indicate that the radical–radical interaction in radical pairs is crucial for magnetic control over the populations of bright and dark excitons on spin sublevels. The magnetic interaction between organic radicals is not only accessible in the ground state of radical molecules, but can also be achieved in the excited state, namely the photogenerated spin-correlated radical pairs, in which the spin–spin exchange and dipolar interactions determine the spin dynamics of two unpaired spins [62]. Feskov *et al.* studied the magnetic-field effect on ion-pair dynamics upon bimolecular photo-induced electron transfer in a solution containing electron donor and acceptor molecules [63]. The triplet yield upon the geminate recombination of photogenerated radical–ion pairs is proven to be dependent on the external field and thus it is reasonable that the photoluminescence from a singlet excited state is also sensitive to magnetic fields for photogenerated radical pairs. As shown in Fig. 4d, the control of photoluminescence produced by the charge recombination of photogenerated radical pairs has been demonstrated in rigid donor–bridge–acceptor molecules under weak magnetic fields by Buck *et al*. [64]. It is found that the critical field for magneto-optical effects is related to the strength of the spin exchange interactions, which can be chemically modified by the rigid donor–bridge–acceptor structure and the solvent environment. Note that the magneto-optical effect on photogenerated radical pairs usually occurs at a relatively low field compared with that for diradical ground states due to weak spin exchange interactions.

### Magneto-optical effects via triplet–triplet pairs

Singlet fission is a spin-allowed excited-state process in which a photogenerated singlet exciton is converted into a spin-correlated triplet pair in organic solid-state materials. The spin correlation between the two paired triplet excitons is very similar to that in photogenerated spin-correlated radical pairs, except that the spin quantum number of triplet pairs is *s* = 2 and produces more complicated spin manifolds. Magneto-optical effects on triplet pairs generated by singlet fission in an organic crystal were studied by Yago *et al*. [65] and the radical pair model with a modification of the spin Hamiltonian was adopted to analyse the observed magnetic-field effects on steady-state and transient photoluminescence related to triplet pairs produced by singlet fission. Triplet–triplet pairs are high-spin excited states consisting of singlet, triplet and quintet signatures, and the inter-triplet spin exchange energy can be determined by monitoring the photoluminescence intensity at varying magnetic fields [66]. Triplet fusion, also called triplet–triplet annihilation, is the reverse process of singlet fission. Triplet fusion and singlet fission both rely on triplet–triplet interactions, and thus the magnetic-field effect on upconverted photoluminescence from the singlet excited state through triplet fusion is observed [67]. Note that the sign of the magneto-optical effect based on triplet fusion is usually opposite to that for singlet fission, and the effect of triplet fusion is strongly dependent on the excitation power due to its nature of non-linear optics [68]. Ha *et al.* engineered inter-triplet exchange coupling and spin mixing between singlet and quintet triplet–triplet pair states in metal–organic frameworks [69]. They observed an anomalous magneto-optical effect corresponding to an additional resonance between the singlet and quintet states that could yield an increased triplet fusion rate under a magnetic field of 0.14 T at room temperature. The spin dynamics of singlet fission and triplet fusion is determined by the intermolecular distance and molecular geometry within triplet–triplet pairs [70] and the above studies are very useful for exceeding the spin statistical limit in these multi-excitonic processes.

In tetracene and its derivatives (e.g. rubrene), both singlet fission and triplet fusion are efficient at room temperature because the energy of the first excited singlet state is very close to twice the energy of its lowest excited triplet state. Magnetic-field effects on the fluorescence of amorphous and polycrystalline rubrene films were initially investigated by Tarasov *et al*. [71] and they found that the features of the magneto-optical effects in these two films were quite different due to the spin lattice relaxation of the photogenerated triplet–triplet pairs. The close molecular packing enhances the inter-triplet spin interaction and thus boosts the interconversion between the spin sublevels in the triplet–triplet pairs for magneto-optical effects. Very recently, Wang *et al.* studied excited-state magneto-optical effects in high-quality rubrene microcrystals and constructed the rubrene-microcrystal-based magnetometer on an optical chip (Fig. 4g). A very large magneto-photoluminescence compared with that for rubrene film was observed with a maximum magnitude of ∼40% at a low field [72], as shown in Fig. 4h. It allows the construction of on-chip information transducers, such as the magneto-optical modulator and the detector of alternative fields. For instance, a cosine-modulated light signal at 2 kHz was obtained under the alternative field between −500 and +500 μT at 1 kHz, while the reference output remained constant at the noise level of the optical magnetometer (Fig. 4i). The large magneto-photoluminescence effect promotes its magneto-optical and spin-optoelectronic applications, including optical imaging of the magnetic field [73,74] and a magnetic-field-switchable laser by optical pumping of the rubrene–emitter blend [75].

A fascinating feature of triplet–triplet pairs is that some of their spin sublevels are coherent upon photogeneration through singlet fission. Burdett *et al.* measured the oscillation of quantum beats in the decay of delayed fluorescence from crystalline tetracene in the absence of a magnetic field [76] and attributed it to a coherent superposition of triplet pair states that share the singlet character during the incoherent relaxation of the initially excited singlet state. Wang *et al.* studied the quantum beats of correlated triplet–triplet pairs in tetracene crystals upon applying magnetic fields [77] and concluded that triplet–triplet pairs were spatially separated and weakly coupled immediately after singlet fission. The entanglement of singlet-character triplet–triplet pairs is an interesting property that correlates the dissociation into free triplets and the fusion into emissive singlets, as well as their non-radiative decay. The high-spin nature of triplet–triplet pairs is ideal for exploration of the spin properties of bound multi-exciton states, and also for attempts on magneto-optical phenomena towards solid-state quantum communication and computing devices.

## SPIN-DEPENDENT PHOTOCHEMICAL REACTIONS

In general, the conservation of angular momentum should be obeyed across a wide array of chemical processes, which is also true for the photo-induced reactivity of molecular systems, whether it involves energy transfer or electron transfer [14]. A reaction would be spin-allowed if the spin angular momentum space spanned by the reactants intersects that spanned by the products. Similarly, an electronic excited state (triplet) with spin angular momentum that is different from that of the ground state (singlet) usually has a long lifetime; meanwhile, it is more likely to undergo photoreactions to decouple the electron spins and transfer the angular momentum to the reaction products. The reaction path of the triplet state could be very different from that for singlet [78] and the photoreaction strongly depends on the singlet/triplet ratio in excited states [79], as well as the coupling strength between the singlet and the triplet manifolds [80]. For instance, in the reaction for photodynamic therapy, a triplet excited state of a photosensitizer is required to produce reactive oxygen species, including the superoxide anion through electron transfer (Type I) and the singlet oxygen reaction by energy transfer (Type II) [81]. Metal-containing photosensitizers are attractive because the strong spin–orbit coupling of heavy metal centers is beneficial for intersystem crossing from photogenerated singlet to triplet excited states. The magnetic-field-boosted generation of reactive oxygen species was also observed in liquid-phase solutions and in living cells [8], which indicates the spin selectivity and spin-related properties of photochemical processes.

The spin–orbit coupling-induced reactivity in photochemistry represents an important type of spin catalysis and this concept was introduced by Minaev and Ågren on the grounds of results from quantum chemical calculations [82]. Spin catalysis describes the spin-related chemical phenomenon in which the reaction is promoted by overcoming spin prohibition or the activation barrier is lowered through spin decoupling induced by a paramagnetic catalyst. It has been recently demonstrated in the magnetic enhancement of electrocatalytic water oxidation in alkaline media [83], in which the field favors the parallel alignment of oxygen radicals during the formation of the O–O bond in oxygen molecules with a triplet ground state. The magnetic nature of the catalysts is strongly correlated with the observed magnetic-field effect on the oxygen evolution reaction (OER). Ren *et al.* used the ferromagnetic ordered catalysts to polarize the spins in the electron-transfer step of the OER under a magnetic field and investigated the spin selection and spin-polarized kinetics of the electrocatalytic OER [84]. They further found that the spin-enhanced effect for the OER could be realized when the domain walls in the ferromagnetic catalysts were reformatted into a single magnetic domain region under an applied field [85]. Their studies have addressed the origin of spin catalysis for the OER on ferromagnetic catalysts, and filled the missing gap between the magnetization of ferromagnetic catalysts and the spin-selective reaction pathway in electrocatalytic water splitting. The concept of spin catalysis was further demonstrated with graphene belts in an oxygen reduction reaction (ORR) and organic synthesis by Tian *et al.* and the reaction kinetics was also studied using the *in situ* ESR technique [86]. The above examples show that electron spin plays an important role in catalytic reactions, and holds great potential for boosting both reaction activity and product selectivity. Spin-enhanced electrocatalytic reactions have been further explored in oxygen reduction, methanol oxidation and carbon dioxide reduction. As spin-electrocatalysis has been recently reviewed elsewhere [87–91], we focus on spin-dependent photochemical reactions based on the spin-related properties of the excited state in this section.

### Triplet-sensitized photoreactions

**Figure 5.**
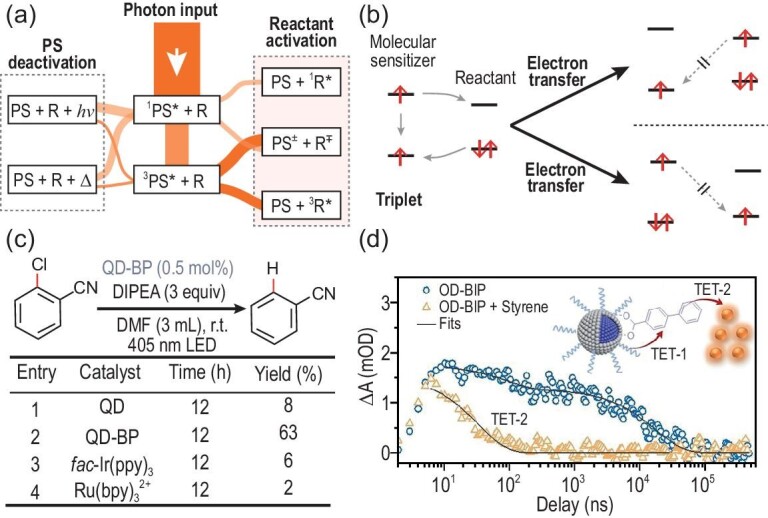
(a) Schematic illustration of the pathways of photogenerated excited states in the photosensitizer (PS) and the reactant (R) for organic transformations. (b) Schematic illustration of the electron transfer from the triplet excited state of the molecular sensitizer to the reactant. Reproduced from Ref. [93] with permission. (c) Photo-reductive dehalogenation of aryl chlorides using various photosensitizers, and the product yields after 12 hours. (d) Excited-state kinetics for the ZnSe/ZnS QDs and biphenyl ligands without and with styrene added. Inset represents the triplet energy transfer (TET-1) from QDs to biphenyl ligands, and the secondary triplet energy transfer (TET-2) to styrene. Reproduced from Ref. [95] with permission.

The triplet state plays the role of a photoexcitation intermediate in chemical and biological photoreactions [92]. Its paramagnetic nature (*s* = 1) allows the study of these triplet excited-state reactions by using light-induced ESR. Molecular photosensitizers can reach their singlet excited states by the absorption of photons and these singlet states can convert into their triplet excited states by spin-flip intersystem crossing [93]. Note that the first triplet excited state is lower compared with the first singlet state in the energy-level alignment. The spin-antiparallel electrons in the triplet state are not favorable for its deactivation to the ground state by either radiative or non-radiative channels, which is much slower compared with the relaxation of singlet excited states. When the photosensitizer is used for organic transformations, its energy transfer or electron transfer to the reactant is involved in the kinetics of excited states, as shown in Fig. 5a. The competition between intrinsic photosensitizer deactivation and extrinsic reactant activation determines the ratios on the excited-state relaxation pathways as well as the mechanism in photosensitized organic transformations. As shown in Fig. 5b, the triplet states of molecular photosensitizers are more efficient for electron transfer, as they have prolonged lifetimes to sensitize reactants, and back electron transfer is also suppressed due to spin conservation in the long-lived triplet states. In addition, triplet sensitizers can transfer energy to the active triplet states of reactants, as triplet–triplet energy transfer is a spin-allowed process and is superior for specific types of photoreactions.

Semiconducting quantum dots (QDs) are an emerging type of photosensitizer that are widely used in organic transformations and other light-harvesting reactions [94]. The photophysical properties of QDs are very different from those of molecular counterparts, while their spin properties are also more complicated, as the spin quantum numbers are not well defined in QDs. The spin configurations of QD excitons cannot be easily classified into paired and parallel states, which are referred to as bright and dark excitons according to the selection rules of optical transitions. The relaxation of dark states in QDs is prolonged in a similar way to that of triplets in molecular photosensitizers, and back electron transfer is also not favored due to the conservation of angular momentum. The QD photosensitizers can transfer the excited-state energy to either singlet or triplet state in reactants through either Förster- or Dexter-type energy transfer. The Dexter energy transfer can activate the reactant molecules into their triplet states for photoreactions. For instance, Nie *et al.* used low-toxicity ZnSe/ZnS core–shell QDs as the visible-light-driven photocatalysts for organic transformations [95]. The surfaces of the QDs were functionalized with benzophenone ligands that rapidly extract electrons from QDs and form long-lived charge-separated states. As shown in Fig. 5c, the dehalogenation of aryl chlorides (2-chlorobenzonitrile) is thermodynamically challenging and the yields are very low when using unmodified ZnSe/ZnS QDs and metal complexes (fac-Ir(ppy)_3_ and Ru(bpy)_3_^2+^). Notably, the yield was significantly improved to 63% in ZnSe/ZnS QD–benzophenone complexes. ZnSe/ZnS QDs were further used to sensitize the triplets of biphenyl ligands through triplet energy transfer and the secondary triplet energy transfer to styrene reactants was demonstrated for triplet-driven [2 + 2] cyclo-addition reactions, as shown in Fig. 5d. It is obvious that the combination of QDs and molecular photosensitizers holds great potential for addressing the spin-related limitations in triplet-sensitized photoreactions.

As mentioned above, triplet–triplet annihilation, also known as triplet fusion, is an alternative way of utilizing triplet energy through the upconversion of low-energy photons into high-energy ones [96]. The spin properties of triplet–triplet pairs are attractive, and this multiphoton process is useful in terms of both photophysics and photochemistry. It can boost the maximal efficiency of solar energy conversion by harvesting sub-bandgap photons in the solar spectrum. Triplet–triplet annihilation allows visible-light-driven reactions to be triggered by near-infrared light, and also enables ultraviolet-activated photosensitized reactions to be achieved with visible light [97]. Recently, Liang *et al.* reported zinc-doped CuInSe_2_ with an external quantum efficiency for near-infrared-to-yellow optical upconversion reaching 16.7% [98]. The nanocrystals were merged with photoredox catalysis, which were used for efficient near-infrared-driven organic synthesis and polymerization. They found that the broadband light absorption of nanocrystals benefits very rapid reactions for high-added-value chemical transformations under indoor sunlight. It is expected that the spin properties of triplet–triplet pairs may play a crucial role in these photochemical processes and the reactions may be sensitive to the magnetism of photocatalysts and/or the application of external magnetic fields.

### Spin-enhanced photocatalysis

Enhancing photocatalytic performance by applying a magnetic field has been experimentally demonstrated for decades [99]. It was not related to the spin properties of reaction intermediates in photocatalysis at the very beginning because the Zeeman energy under a finite field strength is not enough to alter the band structure of catalysts. External fields can facilitate the separation of photogenerated carriers and inhibit the recombination of electrons and holes due to the Lorentz force on charged particles [100]. The Lorentz force can also modulate the mass transfer process, promote the adsorption of reactive ions on the catalyst and thus lead to improved photocatalytic activity [101]. Further studies reveal that the spin-related behavior is critical in determining the photocatalytic performance of ferromagnetic catalysts. For instance, Li *et al.* measured the magnetoresistance in α-Fe_2_O_3_/reduced graphene oxide hybrid nanostructures and improved their photocatalytic performance under a magnetic field [102]. They found that the carriers could transfer from α-Fe_2_O_3_ to reduced graphene oxide more easily under the applied field due to the negative magnetoresistance, providing more charge carriers on the active sites to participate in the photocatalytic process. Besides, an Au-supported Fe_3_O_4_/N-TiO_2_ superparamagnetic photocatalyst was used for photocatalytic overall water splitting under a magnetic field [103]. Strong local magnetic flux was induced in the catalysts to greatly prolong the exciton lifetime, which was attributed to both the Lorentz force and spin-polarization effects.

**Figure 6.**
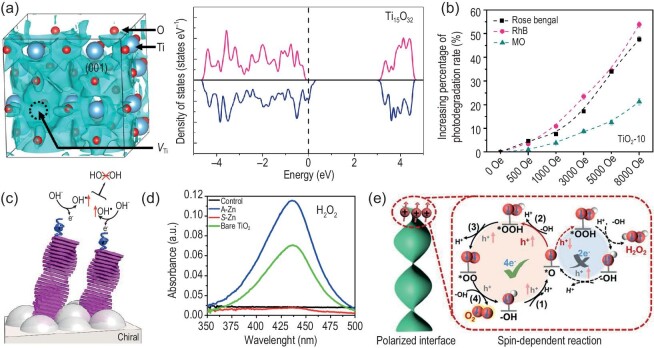
(a) Left: (001)-planar 3D spatial distributions of spin polarization in defected TiO_2_. Right: calculated total density of states in defected TiO_2_. (b) Increasing percentage of photodegradation rates for the Ti_0.936_O_2_ (TiO_2_-10) catalyst under a magnetic-field strength of 0–8000 Oe. Reproduced from Ref. [105] with permission. (c) Illustration of the two spin-parallel OH• interaction on a triplet surface that forbids the production of H_2_O_2_. (d) UV–vis absorption spectra for the H_2_O_2_ production measured from the used electrolyte in the photoelectrochemical cells with bare TiO_2_ and TiO_2_ electrodes coated with self-assembled Zn-porphyrins of either achiral (A-Zn) or chiral (S-Zn). Reproduced from Ref. [111] with permission. (e) Schematic illustration of the spin-polarized surface reaction of photocatalytic O_2_ production on the atomic-level chiral ZnO catalyst. Reproduced from Ref. [113] with permission.

Spin-enhanced photocatalysis does not even require long-range magnetic order in the catalysts, i.e. the spin property of active sites or their short-range magnetic order is essential to manipulate the electron spins for photocatalysis. For instance, TiO_2_ is a nonmagnetic semiconductor for photocatalytic reactions [104] but its metal vacancies may carry spins. Pan *et al.* reported the manipulation of electron-spin polarization in TiO_2_ by tuning the concentration of Ti vacancies (Fig. 6a) and found that the photocatalytic performance of defected TiO_2_ is closely related to the degree of spatial spin polarization [105]. As shown in Fig. 6b, the external magnetic field could further improve the best photocatalytic performance of the Ti_0.936_O_2_ catalyst, which was attributed to the enhanced electron-spin parallel alignment to facilitate the processes of charge separation and surface reaction. Similarly, the concentration of surface oxygen vacancies was precisely controlled in the BaTiO_3_ perovskite and its photocatalytic activity toward N_2_ fixation, related to the spin states of the active surface sites, was largely improved under a magnetic field [106]. Spin-enhanced catalysis can also be achieved by doping magnetic centers in the catalyst. The manipulation of spin-polarized electrons in CsPbBr_3_ was realized by doping Mn cations and the photocatalytic CO_2_ reduction reaction efficiency was boosted by applying an external magnetic field [107]. Gong *et al.* reported manipulation of the spin state of Co centers in covalent organic frameworks by changing the oxidation state of Co in the porphyrin [108]. The spin-state transition in Co centers regulates the photocatalytic performance, and the *s* = 0 state exhibits higher activity and higher selectivity. Huang *et al.* proposed that the dynamic spin-state transition could be chemically manipulated in the Fe–Pt Hofmann clathrates and used it for studying the spin-related structural–catalytic relationship [109]. It was found that only the photocatalytic ORR occurred on the high-spin state of Fe(II), and both the ORR and the water oxidation reaction took place in the photocatalytic synthesis of H_2_O_2_ for the low-spin state. The manipulation of spin states on active sites is not only crucial for improving photocatalytic performance, but also offers exciting possibilities for precise control over photocatalytic reaction pathways.

Chirality is another way to introduce spins in photocatalysts for tailored spin-polarized catalysis according to the CISS effect [110]. As shown in Fig. 6c, the chiral organic semiconductors from helically aggregated dyes were used as the photosensitizers in a photoelectrochemical cell to eliminate H_2_O_2_ formation in the production of hydrogen through water splitting [111]. When the spins of the two electrons are aligned in parallel with each other on the catalyst, the spin interactions of two OH• radicals restrict the formation of H_2_O_2_ and facilitate the production of triplet O_2_. As shown in Fig. 6d, the electrolyte obtained from the bare TiO_2_ and TiO_2_ functionalized with achiral dyes exhibited an absorption peak corresponding to the reaction of H_2_O_2_ and the *o*-tolidine indicator, while no detectable amount of H_2_O_2_ was observed for TiO_2_ with the chiral molecules. Furthermore, Chiral covalent organic frameworks were used for tuning the active sites of coordinated Cu ions for visible photocatalytic hydrogen evolution from water [112]. Ai *et al.* used the spin selectivity effect in an atomic-level chiral ZnO structure to promote the performance of photocatalytic O_2_ production [113]. The spin-polarized carriers produced by the chiral structure not only prolonged the carrier lifetime, but also favored the formation of triplet O_2_ species instead of singlet byproducts, as shown in Fig. 6e. Chiral catalysts provide a feasible platform for investigating spin-related phenomena in photocatalysis, while the manipulation of spin states on photocatalysts can also be useful for the asymmetric synthesis of chiral compounds. Chiral metal–halide perovskite nanocrystals, in which spins could be readily controlled, were used for the asymmetric organic synthesis of N–C axially chiral heterocycles under visible-light activation by Mishra *et al*. [114]. Moreover, the enantioselective synthesis of helical polydiacetylene was realized by the application of linearly polarized light with a parallel/antiparallel magnetic field in the photochemical reaction [115]. The use of circularly polarized light could even induce the chiral fragmentation of an achiral planar molecule [116] and the relationship between the circular polarization of photons and the chemical chirality of molecules is probably related to the spin selections in both of them [117].

## CONCLUSION AND OUTLOOK

In conclusion, spin photochemistry is not a recent concept but definitely needs more research attention, as evidenced by the extensive investigation into spin-related excited-state phenomena over the past several decades. As outlined in this review, the recent breakthroughs have inspired the research community related to this field, particularly in the realm of light-induced magnetic properties, excited-state magneto-optical effects and spin-dependent photochemical reactions. We regret that many exciting advances in spin photochemistry have not been covered here due to the limits of our knowledge and the length of this review article. Nonetheless, it is important to highlight the interdisciplinary nature of spin photochemistry, which encourages groundbreaking collaborations among chemists, physicists, materials scientists, biologists and experts from other relevant disciplines.

Extant challenges and inherent limitations in the development of spin photochemistry include the following issues. Comprehensive theoretical models for incorporating spins and photons in chemical processes are still missing, particularly in the presence of a magnetic field [118]. Spin-related excited-state processes in general share the same underlying physical picture according to the notion of symmetry in spins and lights and chemicals. A universal picture can describe their relationships and is therefore essential for the fundamentals of spin photochemistry. Meanwhile, the state-of-the-art spectroscopic techniques are required to detect photogenerated spin states with high spatial and temporal resolutions [119], especially *in situ*/*operando* measurements for spin-dependent photochemical reactions. The accurate characterization of electron-spin coupling in photochemical molecular systems is also crucial for either strong chemical bonds or weak intermolecular interactions [120]. The materials synthesis of high-spin electronic configuration with unusual magnetic and electronic/optical properties is attractive [121] and needs to be further explored both theoretically and experimentally for the purpose of spin photochemistry [122].

In light of the broad range of possible future applications for spin photochemistry, we would like to put our emphasis on the following three directions:

- Light-induced magnetic properties: they could lead to the design of smart materials for robotics under light and magnetic fields. Magnetically actuated shape changes in soft materials are highly attractive because a magnetic field can safely penetrate most materials, including biological tissues. In contrast, photo-switching in light-responsive materials offers high reversibility and wavelength selectivity. Combining magnetic fields and light irradiation to control the shape change of soft materials is unique and particularly fascinating [123]. Such smart materials could assist in a wide range of productive functions, including robotics and the communication with living organisms. The magnetic and optical control modalities can also be combined in droplet systems that support the management of chemical reactions [124], holding great potential for studies on artificial cells and bio-mimic systems.
- Excited-state magneto-optical effects: they could be useful in the optical control of spin states for information technology. The photogenerated electron–hole pairs can serve as correlated spin-qubit pairs, whose spin dynamics are of particular interest in information technology. Especially, molecular excited states can host multiple spin qubits that have promising properties for quantum information science [125]. Although it is less developed than some of the other qubit platforms such as superconducting and trapped-ion qubits, photochemical molecular systems can serve as spin-qubit arrays taking advantage of excellent chemical versatility, and the highly tunable magnetic, optical and electronic properties of qubits. Besides, the facile self-assembly strategies of molecular materials benefit the fabrication of large ordered qubit arrays [126].
- Spin-dependent photochemical reactions: they would allow the magnetic control of photochemical and biochemical synthesis. Magnetic perturbations can produce observable, and sometimes dramatic, effects on reaction rates, pathways and product yields in photochemical reactions. External magnetic fields (permanent or oscillating) and internal fields (e.g. spin–orbit coupling, hyperfine interaction) can help in overcoming the spin selection rules for excited states as reactive paramagnetic intermediates. Thereby, utilizing the spin-sensitive process offers possibilities for the magnetic control of photochemical synthesis [127]. The reaction pathways involving electron-transfer processes or radical intermediates can be spin-catalysed and/or spin-selected. Spin-state control is also valid in biochemical processes, including enzyme-catalysed DNA synthesis and polymerase chain reactions, which implies the broad potential for magnetic fields to influence and control complex chemical systems.

We hope this review will be well received by the experts in photochemistry and spin chemistry, as well as the scientists who are interested in this emerging field, and aspire to pave the way for fruitful discussions and collaborations towards future development of spin photochemistry. With the ever-growing expertise and enthusiasm in spin photochemistry, we are confident that a wealth of innovative research works and scientific discoveries on spin-related excited-state phenomena will emerge both in China and across the globe in the near future.
